# Mental health monitoring in 5G Edge-Enabled Cognitive IoT with Temporal Shift Transformer and integrated Stackelberg Game Theory and Nomadic People Optimizer

**DOI:** 10.1038/s41598-025-26959-1

**Published:** 2025-12-18

**Authors:** M. Pavithra, G. Ramya

**Affiliations:** School of Computer Science Engineering and Information Systems, Vellore Institute of Technology, Vellore, Tamilnadu 632014 India

**Keywords:** Mental health monitoring, 5G, Cognitive IoT, Temporal Shift Transformer, Stackelberg Game Theory, Nomadic People Optimizer, Engineering, Mathematics and computing

## Abstract

This research proposes an innovative framework for mental health monitoring in 5G Edge-Enabled Cognitive internet of things (IoT) environments, integrating Stackelberg Game Theory and the Nomadic People Optimizer (NPO) algorithm. The temporal shift transformer is introduced as a key component for effective prediction of mental health. The Stackelberg Game Theory ensures strategic decision-making between the central authority and decentralized agents, optimizing resource allocation and enhancing the overall system’s performance. The Nomadic People Optimizer algorithm contributes to the efficiency of the decision-making process, providing an adaptive and dynamic solution for personalized mental health monitoring. The framework aims to address the challenges associated with nomadic lifestyles, leveraging 5G edge capabilities for real-time data processing and analysis. Personalized recommendations are provisioned based on the insights derived from cognitive processing, offering tailored interventions during critical mental health situations. According to experimental data, the suggested framework outperforms baseline models like CNN, GRU, and ResNet-50 + LSTM by achieving 96.38% accuracy, 96.2% F1 score, and 97.2% specificity. Additionally, real-time alert creation with an end-to-end latency of less than 46 ms is made possible by the integration of 5G edge computing, guaranteeing prompt mental health treatments. The proposed approach demonstrates promising results in terms of accuracy, adaptability, and scalability, showcasing its potential to revolutionize mental health care for nomadic populations within the evolving landscape of cognitive IoT and 5G technologies.

## Introduction

A person’s mental health, which includes their psychological, emotional as well social facets of life, is a vital part of their total well-being. The prevalence of psychological illness globally has garnered increasing attention due to their profound impact on individuals, families, and societies. According to the World Health Organization (WHO), psychological illness impact one in every four persons on the planet through certain way of their lives, resulting in a serious public health issue^1^. Mental health problems manifest in various forms, which comprises of mood sicknesses, anxiety conditions, psychotic disorders, and substance use syndromes, contributing to substantial disability and reduced quality of life.

The multifaceted nature of mental illness underlines the complexity of their etiology, involving the combination of biological, environmental, and genetic factors^2^. The global burden of mental health problems is substantial, with an estimated 450 million people currently suffering from such conditions^1^. The societal implications extend beyond individual suffering, affecting productivity, economic resources, and healthcare systems worldwide. Mental health problems are also associated with increased mortality rates, with individuals experiencing mental disorders often facing elevated risks of chronic physical conditions^3^.

The stigma surrounding mental health further exacerbates the challenges faced by individuals seeking help. Despite advancements in mental health awareness, a significant treatment gap exists, and many individuals with mental health problems go untreated or face delays in accessing appropriate care^4^. Additionally, the COVID-19 pandemic has amplified the global mental health crisis, with bigger stressors, economic uncertainties and social isolation contributing for surge in mental health challenges^5^.

Efforts to address mental health problems require a comprehensive and integrated approach, encompassing prevention, early intervention, and accessible treatment options. Research in mental health is essential for advancing the understanding for protective factors, risk factors, and effective interventions. The prevalence over mental health disorders globally underscores the pressing need for effective monitoring systems that can provide timely interventions and support. With advancements in technology, particularly concentrating over Artificial Intelligence(AI), IoT (Internet of Things) and data analytics, there exists a unique opportunity to revolutionize the landscape of mental health monitoring.

In response to this pressing concern, the integration of technology into mental health care has gained traction, ushering in a new era of continuous and personalized monitoring. This evolution aligns with the principles of precision medicine, acknowledging the unique characteristics of individuals and tailoring interventions accordingly^6^. The initiation of wearables, smartphones, much other connected technologies has facilitated the pool of large data collected on relevant to mental health, encompassing physiological, behavioral, and environmental indicators.

The EEG, among various clinical data, provides insights into profound aspects of human cognitive function^7,8^. This recording captures the rhythmic and spontaneous electrical impulses generated by brain cells over scalp. From the period when the exploration of monkey brain and keeping records of brain EEG signal, researchers were delved into a correlation of brain function over mental disorders, employing EEG data as a valuable resource. This data-rich landscape has paved the path for creating advanced monitoring systems capable of providing real-time insights into an individual’s mental health status. The potential of such systems is further amplified by the deployment of edge computing and 5G technologies, enabling rapid and decentralized data processing^9^.

However, the dynamic and nomadic nature of modern lifestyles poses unique challenges to mental health monitoring systems. To address these challenges, innovative frameworks that incorporate advanced algorithms and decision-making models are essential. The convergence of Stackelberg Game Theory and optimization algorithms, such as the Nomadic People Optimizer (NPO)^10^, grasps assurance on improving adaptability and usefulness of mental health monitoring in dynamic environments.

Although wearable sensor integration, IoT-based health monitoring systems, and deep learning methods for mental disorder prediction have all been studied in the past, these studies usually have three main drawbacks:

### Centralized architectures

Since most current systems mostly rely on cloud-based processing, they are not appropriate for real implementations, mobile based mental health applications due to excessive latency and privacy issues.

### Temporal modeling

Even if they are employed for sequential data, traditional models such as CNN, LSTM, or GRU perform poorly in low-resource edge contexts and have trouble capturing long-range dependencies.

### Static optimization approaches

Prior work’s optimization techniques are frequently inflexible and unable to adjust to dynamic shifts in user context or network constraints, especially for nomadic populations with sporadic access.

To fill these shortcomings, however, our suggested architecture offers a unique fusion of three technologies:

- High-accuracy, paralleled temporal modeling of EEG signals is made possible by the Temporal Shift Transformer (TST), which outperforms recurrent architectures while preserving computing economy for edge deployment.
- In order to support strategic leader-follower relations among central and edge devices on basis of system limits and user needs, Stackelberg Game Theory is integrated for adaptive resource allocation.
- The integration of the Nomadic People Optimizer (NPO) guarantees dynamic optimization and customization, adjusting model behavior to evolving lifestyles and behavioral patterns.

## Related works

Valsalan et al.^11^ presented an IoT-based health monitoring system, focusing on critical reviews and advancements in health monitoring. Durán-Vega et al.^12^ proposed the system of IoT tailored monitoring elderly adults’ health monitoring from remote, using mobile application through wearables for comprehensive health data collection. In order to improve security, Ali et al.^13^ presented an industrial Internet of Things (IoT) along with blockchain-based securely searchable encryption method for healthcare systems that incorporates neural networks. Almaiah et al.^14^ contributed a unique hybrid reliable de-centralized authentication and data retention strategy for Internet of Things-based digital healthcare Cyber-Physical Systems (CPS), aiming to enhance authentication and data integrity in the context of healthcare IoT.

The innovative applications of artificial intelligence (AI) and machine learning (ML) systems in health-care are explored to enhance smart health monitoring and clinical practice. Sujith et al.^15^ conducted a methodical research focusing on smart health monitors utilizing AI and deep learning, highlighting the potential of these technologies in revolutionizing healthcare delivery. Cimtay and Ekmekcioglu^16^ investigated the efficacy of pre-trained CNN (Convolutional Neural Networks) for EEG emotion classification across different subjects and datasets, showcasing the versatility and transferability of deep learning models in mental health assessment. Aldahiri et al.^17^ delved into the trends of applying Internet of Things (IoT) coupled with ML for health prediction systems, elucidating the synergy between IoT-generated data and predictive analytics for proactive healthcare management. Lastly, Ed-Driouch et al.^18^ addressed the challenges associated with integrating ML into clinical practice through a hybrid human-machine intelligence approach, emphasizing the importance of collaborative decision-making processes in healthcare settings.

Mirjalali et al.^19^ focused on the potential applications of wearable sensors for remote health monitoring, specifically exploring their role in the early diagnosis of COVID-19. The study delves into the technological advancements in wearable sensors and their capacity to contribute to the proactive and remote monitoring of health, with a particular emphasis on detecting early signs of infectious diseases such as COVID-19. Hammad et al.^20^ contributed to the field of IoT healthcare applications by proposing deep learning models for arrhythmia detection. Their work demonstrated the integration of advanced machine learning techniques for the accurate identification of arrhythmias, leveraging the capabilities of IoT in healthcare settings. This research addresses the critical need for efficient and automated arrhythmia detection to enhance patient care.

Nancy et al.^21^ presented an IoT-cloud-based smart healthcare monitoring system designed for heart disease prediction through deep learning. This study explores the integration of IoT and cloud computing to develop an intelligent healthcare monitoring system. By employing deep learning algorithms, the authors aim to predict heart diseases, demonstrating the potential of advanced technologies in revolutionizing predictive healthcare models. Haq et al.^22^ introduced DACBT, a deep learning approach for the classification of brain tumors using MRI data in the IoT healthcare environment. This research focused on leveraging deep learning techniques for the automated and accurate classification of brain tumors, showcasing the potential of IoT in enhancing diagnostic capabilities in the healthcare sector.

An innovative Internet of Things (IoT) and fog computing-based observing system designed specifically for cardiac patients. The study addresses the need for continuous and efficient monitoring of cardiovascular health by leveraging advanced technologies. The proposed system integrates IoT devices and fog computing to create a decentralized and responsive framework capable of real-time data processing.

The necessity of distributed collaboration between models and devices has been highlighted by recent developments in Edge General Intelligence, especially in low-latency, high-stakes scenarios like health monitoring. In order to address issues with adaptability, trust and resource-aware scheduling, Luo et al.^23^ presented the Multi-LLM architecture, which allows orchestration of numerous large language models across edge and cloud tiers. A trust-based orchestration layer is introduced in their approach, which dynamically chooses and routes inference tasks according to data sensitivity, device capabilities, and model relevance. Our study incorporates game theoretic orchestration, current EEG processing and temporal modeling (TST) over 5G edge nodes to apply comparable ideas to mental health intelligence, in contrast to their focus on block-chain consensus and safe transactions in cognitive radio networks. Both systems, however, strive for reliable, decentralized, and effective processing on wireless Internet of Things networks.

## Methodology

To tackle the multifaceted complexity of real-world mental health monitoring in mobile and edge-enabled cognitive IoT contexts, the Temporal Shift Transformer (TST), Stackelberg Game Theory, and Nomadic People Optimizer (NPO) have been carefully integrated. Every part aims to meet a different but related system requirement.

1. A lightweight yet effective method for simulating non-linear, long-range temporal dependence in behavioral and EEG data is the Temporal Shift Transformer (TST), which enables precise, low-latency inference right on edge devices. TST eliminates sequential bottlenecks and facilitates parallel processing, in contrast to RNN-based models.
2. Hierarchical resource allocation and decision-making between a central orchestrator (like an edge server or cloud provider) and distributed agents (like wearables) are governed by Stackelberg Game Theory. This ensures fairness and logical participation by enabling the system to adaptively assign energy, bandwidth and upgrade schedules depending on both system-level goals (like minimizing latency) and device on local demands (like battery saving).
3. In order to handle dynamic optimization in real-world scenarios, including user mobility, signal fluctuation, and erratic connectivity, Nomadic People Optimizer (NPO) is included. The non-stationary, multi-objective character of mental health settings, where optimization criteria (such as accuracy, energy, and delay) change over time and across users, is ideally suited for its population-based, migration-inspired search.

**Fig. 1:**
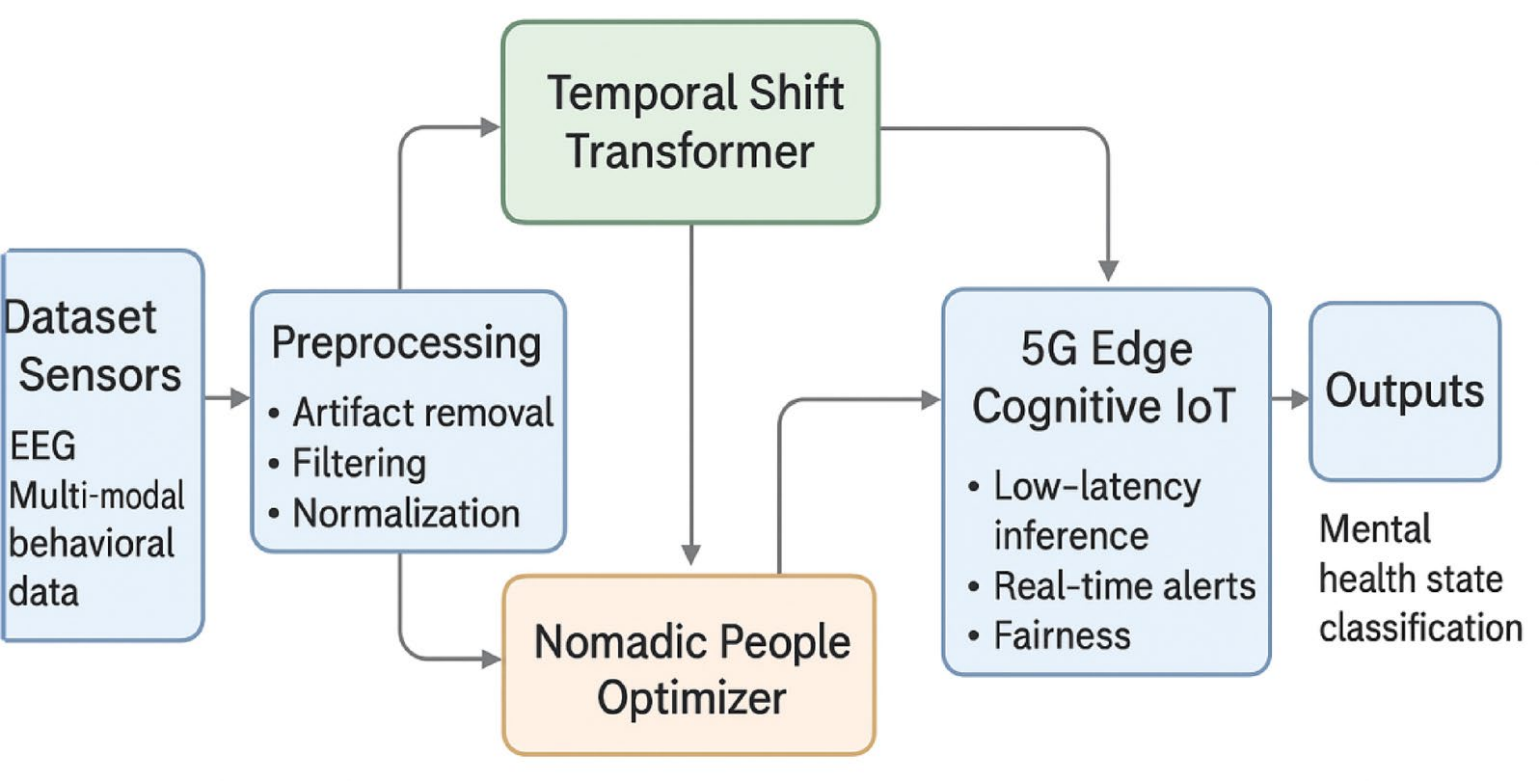
Flow of the proposed work.

The research methodology utilized in this study involves the expansion and application of an innovative Mental Health Monitoring framework designed for 5G Edge-Enabled Cognitive Internet of Things (IoT) environments. The core components of the framework include the Temporal Shift Transformer, Stackelberg Game Theory, and the Nomadic People Optimizer (NPO) algorithm. The Temporal Shift Transformer serves as a key element for effective mental health prediction, capturing temporal patterns within the collected EEG signals. Stackelberg Game Theory is integrated to facilitate strategic decision-making between a central authority and decentralized agents, optimizing resource allocation for enhanced system performance. The Nomadic People Optimizer algorithm contributes to the adaptive and dynamic nature of the decision-making process, tailoring mental health monitoring to individual needs. The framework leverages the capabilities of 5G edge computing for real-time data processing and analysis, addressing challenges associated with nomadic lifestyles. Personalized recommendations are generated based on insights derived from cognitive processing, enabling tailored interventions during critical mental health situations. The methodology involves rigorous testing and validation against existing algorithms, ensuring the effectiveness and superiority of the proposed framework in predicting and monitoring mental health conditions in dynamic IoT environments. Figure 1 shows the proposed work flow.

### Dataset

The MODMA dataset, a multi-modal open dataset utilized in investigating psychological disorders, contains EEG signals obtained from two distinct sources Ksibi et al^24^. Firstly, it encompasses data from a conventional elastic cap containing 128-electrode, standard EEG recording kit utilized in clinical as well research purposes. This cap is positioned above the member’s scalp, featuring 128 electrodes designed in relevance to International 10–10 system. Those 128 electrodes capture EEG waves produced by human brain, which are then transmitted to an amplifier for amplification and conversion into digital data for subsequent analysis^25^.

EEG signals obtained using two different kinds of devices as in Table 1 are part of the MODMA (Multi-modal Open Dataset for Mental-disorder Analysis) Hawes et al.^26^, which is used in the suggested framework. Depending on the device setup, both modalities produce time-series data in microvolt (µV) amplitude form with typical sampling frequencies between 128 Hz and 512 Hz.

**Table 1:** Input data type.

Device type	Modality	Signal description
Clinical-grade EEG cap	128-electrode EEG	High-resolution EEG recordings applying International 10–10 system
Wearable EEG collector	3-electrode EEG	Low resolution and Portable data from EEG taken on forehead or ear contacts
Auxiliary signals	EOG, timestamps	Eye movement artifacts and temporal references

Additionally, the dataset includes data from novel EEG-collector made of 3-electrode, designed for various other applications. These handy devices are typically worn over forehead or beside ears, featuring 3 electrodes to directly contact human skin to discover EEG generated by brain. These signals are then transmitted over wireless devices and smartphone for recording and further analysis. Furthermore, standard artifact correction methods, like spatial filtering techniques and time-domain signal filtering, has applied towards enhancing excellence of EEG signals, Liu et al^27^.

### Fairness interpretation

#### Age bias observation

Compared to the younger group, accuracy drops by about 2% on older age group. Minimum training data over this group or age basis EEG pattern fluctuation could be the cause of this.

#### Gender fairness

The model’s performance is almost the same for both male and female groups, indicating that there is no discernible gender bias in this dataset.

### Preprocessing

Pre-processing for MODMA dataset encompasses a series of essential steps aimed at enhancing the quality and reliability of EEG signals acquired through both the traditional 128-electrode elastic cap and the innovative wearable 3-electrode EEG collector.

#### Eye movement artifact reduction

The EEG signals obtained from both elastic cap possessing 128-electrodes and the wearable (3-electrode EEG collector) may be affected by artifacts, particularly those induced by eye movements (EOG). To mitigate this, the following equation is applied for eye movement artifact reduction.

1 $$\:{\text{E}\text{E}\text{G}}_{\text{c}\text{l}\text{e}\text{a}\text{n}}\left(\text{t}\right)={\text{E}\text{E}\text{G}}_{\text{r}\text{a}\text{w}}\left(\text{t}\right)-{\upbeta\:}\times\:{\text{E}\text{O}\text{G}}_{\text{r}\text{a}\text{w}}\left(\text{t}\right)$$

Here, $\:{\text{E}\text{E}\text{G}}_{\text{c}\text{l}\text{e}\text{a}\text{n}}\left(\text{t}\right)$represents the artifact-corrected EEG signal, $\:{\text{E}\text{E}\text{G}}_{\text{r}\text{a}\text{w}}\left(\text{t}\right)$ is the raw EEG signal, and $\:{\text{E}\text{O}\text{G}}_{\text{r}\text{a}\text{w}}\left(\text{t}\right)$denotes the raw EOG signal. The parameter β adjusts the influence of the EOG signal on the EEG signal.

#### Lighting level standardization

Variations in lighting conditions during EEG recording sessions can introduce additional noise. To standardize the EEG signals with respect to lighting levels, the following equation is employed.

2 $$\:{\text{E}\text{E}\text{G}}_{\text{s}\text{t}\text{a}\text{n}\text{d}\text{a}\text{r}\text{d}\text{i}\text{z}\text{e}\text{d}}\left(\text{t}\right)=\frac{{\text{E}\text{E}\text{G}}_{\text{c}\text{l}\text{e}\text{a}\text{n}}\left(\text{t}\right)-{\upmu\:}}{{\upsigma\:}}$$

Here, $\:{\text{E}\text{E}\text{G}}_{\text{s}\text{t}\text{a}\text{n}\text{d}\text{a}\text{r}\text{d}\text{i}\text{z}\text{e}\text{d}}\left(\text{t}\right)$represents the standardized EEG signal, µ denotes mean, and σ denotes standard deviation of the artifact-corrected EEG signal. This normalization ensures consistent signal representation across varying lighting conditions, enhancing the reliability of subsequent analyses.

Signal Filtering is a crucial step in the preprocessing of EEG signals to enhance the quality and extract relevant information. This process involves both Time-Domain Signal Filtering and Spatial Filtering Techniques.

#### Time-domain signal filtering

The EEG signals are initially standardized to a common scale to facilitate consistent processing across different sessions or subjects. The standardized EEG signal, denoted as $\:{\text{E}\text{E}\text{G}}_{\text{s}\text{t}\text{a}\text{n}\text{d}\text{a}\text{r}\text{d}\text{i}\text{z}\text{e}\text{d}}\left(\text{t}\right)$, is then subjected to time-domain filtering. The filtering operation is denoted as

3 $$\:{\text{E}\text{E}\text{G}}_{\text{f}\text{i}\text{l}\text{t}\text{e}\text{r}\text{e}\text{d}}\left(\text{t}\right)=\text{f}\text{i}\text{l}\text{t}\text{e}\text{r}\left({\text{E}\text{E}\text{G}}_{\text{s}\text{t}\text{a}\text{n}\text{d}\text{a}\text{r}\text{d}\text{i}\text{z}\text{e}\text{d}}\right(\text{t}\left)\right)$$

The specific filter function applied depends on the chosen algorithm or method, and it is tailored to attenuate noise, artifacts, or unwanted frequency components.

#### Spatial filtering techniques

Spatial filtering is employed following time-domain filtering, for additional refinement of EEG signals. The filtered signal, denoted as $\:{\text{E}\text{E}\text{G}}_{\text{f}\text{i}\text{l}\text{t}\text{e}\text{r}\text{e}\text{d}}\left(\text{t}\right)\:$undergoes spatial filtering denoted as

4 $$\:\:{\text{E}\text{E}\text{G}}_{\text{s}\text{p}\text{a}\text{t}\text{i}\text{a}\text{l}}\left(\text{t}\right)=\text{s}\text{p}\text{a}\text{t}\text{i}\text{a}\text{l}\_\text{f}\text{i}\text{l}\text{t}\text{e}\text{r}\left({\text{E}\text{E}\text{G}}_{\text{f}\text{i}\text{l}\text{t}\text{e}\text{r}\text{e}\text{d}}\right(\text{t}\left)\right)$$

Spatial filtering techniques aim to enhance the spatial characteristics of the EEG signals, emphasizing relevant spatial patterns while suppressing noise and interference.

#### International 10–10 system

In the context of the 128-electrode elastic cap, the EEG signals are aligned according to the International 10–10 System. This system provides a standardized set of electrode placements on the scalp. The alignment process, represented by the equation.

5 $$\:{\text{E}\text{E}\text{G}}_{\text{a}\text{l}\text{i}\text{g}\text{n}\text{e}\text{d}}\left(\text{t}\right)=\text{a}\text{l}\text{i}\text{g}\text{n}{(\text{E}\text{E}\text{G}}_{\text{s}\text{p}\text{a}\text{t}\text{i}\text{a}\text{l}}\left(\text{t}\right))$$

involves adjusting the spatial coordinates of EEG electrodes to adhere precisely to the predefined positions specified by the International 10–10 System. This alignment ensures consistency and comparability across different recordings and subjects.

#### Wearable EEG collector

For the wearable 3-electrode EEG collector, the EEG signals undergo a similar alignment process. The equation.

6 $$\:{\text{E}\text{E}\text{G}}_{\text{w}\text{e}\text{a}\text{r}\text{a}\text{b}\text{l}\text{e}}\left(\text{t}\right)=\text{a}\text{l}\text{i}\text{g}\text{n}{(\text{E}\text{E}\text{G}}_{\text{s}\text{p}\text{a}\text{t}\text{i}\text{a}\text{l}}\left(\text{t}\right))$$

signifies the alignment of EEG signals acquired through the wearable device. Despite the reduced number of electrodes, accurate alignment remains crucial for preserving the spatial information captured by the limited set of electrodes. The alignment procedure optimizes the correspondence between the wearable EEG electrode positions and their intended locations on the scalp.

#### Consistency checks

Consistency checks are performed to assess the reliability and uniformity of EEG data across different sessions. The Consistency Score is calculated by determining the number of sessions with consistent EEG patterns and dividing it by the total number of sessions. Mathematically, the Consistency Score is represented as

7 $$\:\text{C}\text{o}\text{n}\text{s}\text{i}\text{s}\text{t}\text{e}\text{n}\text{c}\text{y}\_\text{S}\text{c}\text{o}\text{r}\text{e}=\frac{\text{N}\text{u}\text{m}\text{b}\text{e}\text{r}\:\text{o}\text{f}\:\text{C}\text{o}\text{n}\text{s}\text{i}\text{s}\text{t}\text{e}\text{n}\text{t}\:\text{S}\text{e}\text{s}\text{s}\text{i}\text{o}\text{n}\text{s}}{\text{T}\text{o}\text{t}\text{a}\text{l}\:\text{S}\text{e}\text{s}\text{s}\text{i}\text{o}\text{n}\text{s}}\:\:\:\:\:\text{}\:\:$$

Consistency is crucial in ensuring that the acquired EEG signals exhibit stability and coherence across various recording sessions. A higher Consistency Score indicates a more reliable dataset with consistent patterns, adding to the total robustness of further analyses.

#### Quality assessment

Quality assessment focuses on evaluating the validity and integrity of EEG segments within the dataset. The Quality Score has derived through the amount of valid EEG segments and dividing them through entire number of EEG segments. Mathematically, the Quality Score is represented as.

8 $$\:\text{Q}\text{u}\text{a}\text{l}\text{i}\text{t}\text{y}\_\text{S}\text{c}\text{o}\text{r}\text{e}=\frac{\text{V}\text{a}\text{l}\text{i}\text{d}\:\text{E}\text{E}\text{G}\:\text{S}\text{e}\text{g}\text{m}\text{e}\text{n}\text{t}\text{s}}{\text{T}\text{o}\text{t}\text{a}\text{l}\:\text{E}\text{E}\text{G}\:\text{S}\text{e}\text{g}\text{m}\text{e}\text{n}\text{t}\text{s}}\:\:\:\:\:\text{}$$

Valid EEG segments refer to portions of the data that meet predefined criteria for quality, such as minimal artifacts and consistent signal characteristics. A higher Quality Score indicates a dataset with a greater proportion of high-quality EEG segments, ensuring that subsequent analyses are based on reliable and meaningful data.

### Temporal shift transformer (TST) for mental health prediction

**Fig. 2:**
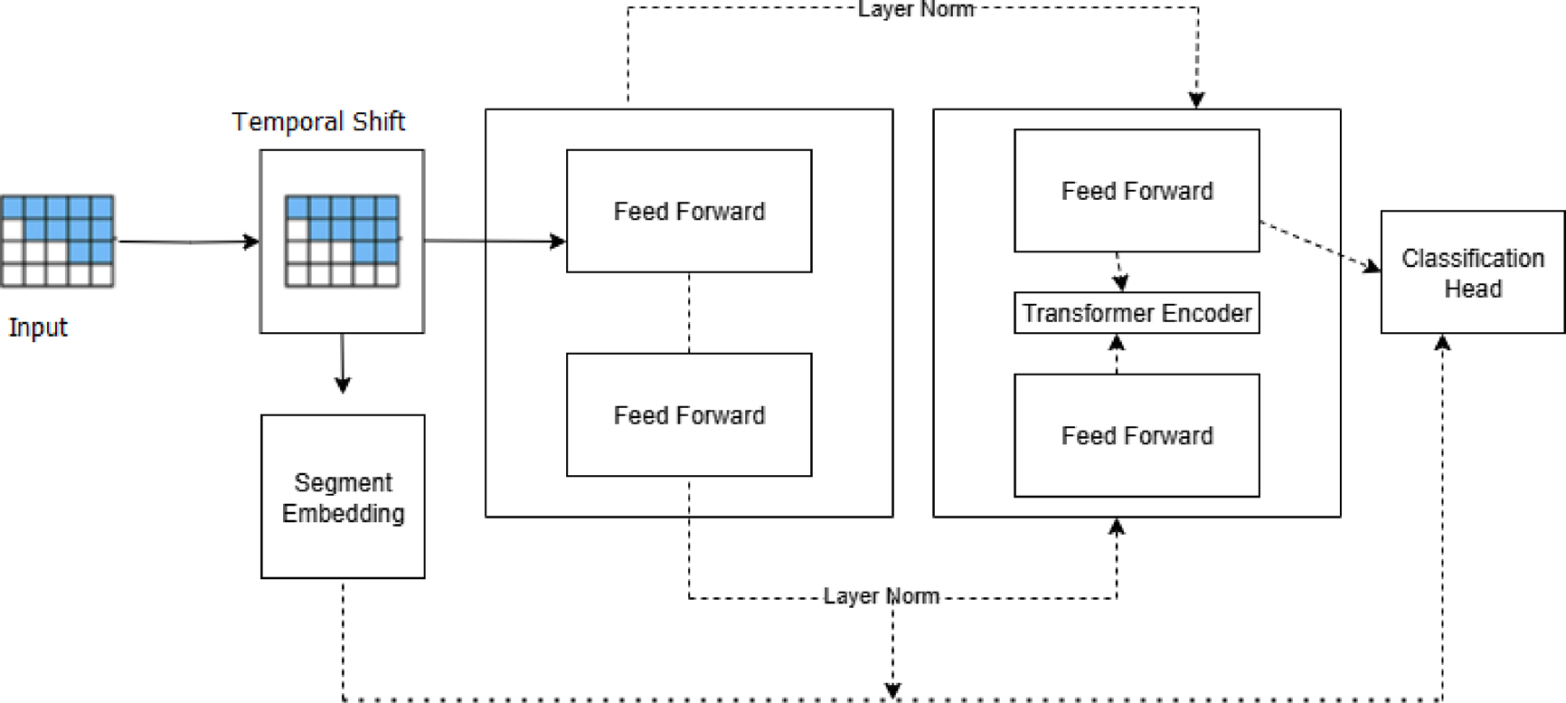
Temporal shift transformer.

The Temporal Shift Transformer (TST) explained in Fig. 2 is introduced as a pivotal component within the proposed framework for effective mental health prediction in the context of Cognitive IoT data. The Temporal Shift Transformer (TST) utilizes EEG signal data from the MODMA dataset for mental health prediction through a multi-step process. The algorithm utilizes the concept of temporal convolution to analyze sequential data over time, making it particularly adept at recognizing patterns and variations in cognitive signals, Wang et al.^28^ .

By integrating effective localized temporal modeling with global context awareness, Temporal Shift Transformer overtakes LSTM/GRU models in time-series mental health analysis. It provides faster and better prediction performance on irregular and long-sequence data. Conventional LSTMs and GRUs analyze sequential data using recurrent operations, which restricts their capacity to represent long-range dependencies and raises computational costs because of the sequential processing. Temporal Shift Transformers (TST), on the other hand, completely eliminate recurrence by enabling temporal interaction between adjacent time steps through a shift mechanism without the need for costly attention computations. As a result, TSTs are quicker and more scalable, particularly for lengthy sequences that are frequently used for mental health monitoring (such as sensor data, sleep, or mood), Uyulan et al.^29^.

### Temporal convolution operation

The Temporal Convolution Operation is a fundamental mathematical operation utilized in the Temporal Shift Transformer (TST) for analyzing EEG sequential data over time. This operation is much essential for capturing temporal patterns along the dependencies within cognitive IoT data, enabling effective mental health prediction.

9 $$\:Y\left[t\right]={\sum\:}_{k=1}^{K}X[t-k].W\left[k\right]+b$$

X[t] represents the input data at a specific time t. This data could encompass various cognitive signals such as brainwave patterns, physiological measurements, behavioral data collected from IoT sensors worn by individuals. The temporal filter weights, denoted as W[k], are parameters that define the characteristics of the filters applied during convolution. These weights determine how the input data is transformed as it passes through the convolutional operation. Each weight corresponds to a specific time step k, influencing the convolution process accordingly. The bias term, represented as b, is a constant parameter added to the output of the convolution operation to introduce a degree of flexibility and enable the model to learn complex patterns in the data. The convolution operation involves sliding a filter kernel (defined by the weights W[k]) across the input data (X[t]) for figuring out the input’s weighted sum within a defined window. This process is repeated for each time step t, resulting in a series of convolved output values. The convolved output values from each time step are summed together; additionally bias value is included to produce this final output Y[t]. This aggregation captures the of-time dependencies along with patterns existent in the input data over a range of time steps K. By applying the Temporal Convolution Operation, the Temporal Shift Transformer can effectively analyze temporal dependencies within cognitive IoT data, facilitating the prediction of mental health states. The convolutional operation allows the model to extract relevant features and patterns from sequential data, enabling accurate and insightful predictions regarding individuals’ mental well-being over time.

### Positional encoding

The positional encoding is computed using sine and cosine functions, which encode positional information into the input data in a continuous and systematic manner. The equations for positional encoding are listed as:

10 $$\:PE(pos,2i)=sin\left(\frac{pos}{{10000}^{2i/d}}\right)$$

11 $$\:PE(pos,2i+1)=cos\left(\frac{pos}{{10000}^{2i/d}}\right)$$

Here, pos signify the position of each element over the sequence, i signify the index of the encoding dimension, and d represents the dimensionality of the positional encoding. These values of positional encoding are then included for input data, providing the model with positional data which guide to recognise this sequential order in data. The sine and also cosine functions used in the positional encoding ensure that adjacent positions have different encoding values, allowing the model to distinguish between different positions within the sequence. Additionally, the positional encoding values are scaled by a factor of $\:{10000}^{2i/d}$, which ensures that the values fall within a suitable range for effective encoding.

### Multi-head attention mechanism

The mechanism of Multi-Head Attention provides efficiency for the model on focusing simultaneously over the various parts in the input sequence. This mechanism is especially effective in capturing complex relationships within temporal data. The equation for the multi-head attention operation is given by:

12 $$\:MultiHead(Q,K,V)=Concat({head}_{1},....{head}_{h}).{W}_{O}$$

Here, Q, K, and V symbolize the input queries, keys, and values, correspondingly. The operation involves splitting these inputs into multiple heads (ℎ), computing attention independently for each head, and then concatenating the results. The weight matrix W_O_ is applied to obtain the final output. The independence of attention computations across multiple heads allows the TST to capture diverse aspects from input system concurrently, enhancing its aptitude towards discern intricate temporal patterns.

### Position-wise feedforward networks

The Position-wise Feedforward Networks (FFN) contributing to the model’s capacity to produce non-linear connection within the temporal data. This FFN operation is expressed by the following equation:

13 $$\:FFN\left(x\right)=max(0,x.{W}_{1}+{b}_{1}).{W}_{2}+{b}_{2}$$

In this equation, x represents the input data, and $\:{W}_{1},{b}_{1},{W}_{2},{b}_{2}$are learnable parameters. The operation involves with activation function rectified linear unit (ReLU) introduced to the linear transformation W1​+b1​, followed by another linear transformationW2​+b2​. This architecture agrees the TST to intricate patterns inside the temporal records by introducing non-linearities through the ReLU activation function. The position-wise nature of the feedforward networks enables the model to adaptively learn and represent complex relationships at different positions within the input sequence, enhancing its overall capability to understand and predict mental health dynamics effectively.

### Stackelberg game theory

The integration of Stackelberg Game Theory in the proposed framework for Cognitive IoT-based Mental Health Monitoring serves as a strategic decision-making mechanism between a central authority and decentralized agents. Stackelberg Game Theory is particularly apt for modelling interactions in scenarios where decision-makers have varying levels of information and authority. In the context of mental health monitoring, this integration addresses the dynamic nature of cognitive IoT environments and the need for strategic resource allocation.

We impose the following realistic constraints, ensuring the tractability and applicability of the game in temporal mental health monitoring. Strategy spaces XXX and Y(x) are non-empty, compact, and convex. UF(x, y), U_F(x, y), UF​(x, y) is strictly concave in yyy for any fixed xxx, and continuous. UL(x, y), U_L(x, y), UL​(x, y) is concave in xxx for fixed yyy, and continuous. The follower’s response may be subject to bounded noise, delay, or limited rationality. Thus (14) is a Stackelberg Equilibrium.

14 $$(x * ,y * )(x^{{ \wedge *}} ,y^{{ \wedge *}} )(x * ,y * )$$

### Central authority role

The central authority, often representing a governing entity or a central server, plays the role of the leader in the Stackelberg Game. It has a global view of the network and possesses information regarding the overall mental health monitoring objectives, resource availability, and system constraints.

15 $$\:{\text{m}\text{a}\text{x}}_{{\text{P}}_{\text{L}}}{\text{U}}_{\text{L}}({\text{P}}_{\text{L}},{\text{P}}_{\text{F}})$$

The central authority aims to maximize its utility U_L_​, which is a function of its own decision variable P_L_ and the decision variable of the decentralized agents P_F_. This utility function encapsulates the leader’s objectives, such as optimizing resource allocation for mental health monitoring.

### Decentralized agents

Decentralized agents, representing individual devices or nodes within the Cognitive IoT network, act as followers in the Stackelberg Game. These agents have limited information about the overall network state but make decisions autonomously based on their local observations and objectives related to mental health monitoring.

16 $$\:{\text{m}\text{a}\text{x}}_{{\text{P}}_{\text{F}}}{\text{U}}_{\text{F}}({\text{P}}_{\text{L}},{\text{P}}_{\text{F}})$$

Each decentralized agent seeks to maximize its utility U_F_, which depends on both the central authority’s decision P_L_ and the agent’s own decision P_F_. The utility function captures the strategic response of agents in optimizing their own objectives within the system.

### Leader-follower dynamic

The central authority, acting as the leader, makes strategic decisions regarding resource allocation, task assignment, and overall network optimization. These decisions are made with the aim of maximizing global objectives, such as system efficiency, accuracy of mental health predictions, and resource utilization. Decentralized agents, as followers, respond to the decisions made by the central authority. Their actions are influenced by the leader’s strategies, and they adapt locally to contribute to the achievement of global objectives. This dynamic reflects the hierarchical nature of the Stackelberg Game. The role of a Leader is to plan model updates, allocates bandwidth, plan for system-wide operations, and makes that the system satisfies energy, latency, and accuracy requirements. The role of the follower is to respond to the central authority’s resource allocation by adjusting local operations (e.g., model precision, update frequency, energy usage). The utility function is as follows,

17 $${\text{U}}_{{\text{L}}} \left( {{\text{P}}_{{\text{L}}} {\text{,P}}_{{\text{F}}} } \right) = \sum\nolimits_{{({\text{i}} = 1)}}^{{\text{N}}} {\left[ {\lambda _{1} .{\text{A}}_{{\text{i}}} \left( {{\text{P}}_{{\;\;{\text{F}}}}^{{\text{i}}} } \right) - \lambda _{2} .{\text{C}}_{{\text{i}}} \left( {{\text{P}}_{{\text{L}}} ,{\text{P}}_{{\;\;{\text{F}}}}^{{\text{i}}} } \right)} \right]}$$

Where,

**A**_**i**_ **( P**^**i**^_**F**_**)**: Mental health prediction accuracy achieved by device i.

**C**_**i**_ **(P**_**L**_, **P**^**i**^_**F**_**)**: Cost (E.g., Energy, delay, bandwidth).

**λ**_**1**_, **λ**_**2**_: Weights for balancing performance vs. cost.

### Dynamic adaptation

The decentralized agents, by responding to the central authority’s decisions, dynamically adapt their behavior based on the evolving network conditions and mental health monitoring requirements. This adaptability is crucial for addressing the changing nature of cognitive data and the nomadic aspects of individuals in the monitored population. The integration of Stackelberg Game Theory ensures a balance between global objectives, set by the central authority for comprehensive mental health monitoring, and local objectives pursued by individual agents to optimize their own performance within the network. The central authority guides the strategic decisions to optimize resource allocation and global objectives, while decentralized agents respond and adapt locally, collectively contributing to the effectiveness and efficiency of mental health monitoring within the dynamic Cognitive IoT environment, Ahmed et al.^30^

### Role of Nomadic People Optimizer algorithm in cognitive IoT-based personalized mental health monitoring

The Nomadic People Optimizer (NPO) algorithm providing adaptive and dynamic solutions for personalized mental health monitoring in Cognitive IoT environments. NPO is a nature-inspired optimization algorithm designed to adapt to dynamic and changing environments. It is particularly well-suited for scenarios where individuals have nomadic lifestyles, as it could animatedly regulate their optimization strategy built over evolving conditions, Alkhdour et al^31^.

#### Fitness function

The fitness function f(x) weighs for performance or excellence of a solution x in the context of mental health monitoring. This function captures the objectives related to personalized monitoring, such as accuracy, reliability, and adaptability.

### Position update

The position update defines how each individual’s position (x_i_) evolves over time.

18 $$\:{x}_{i}(t+1)={x}_{i}\left(t\right)+{V}_{i}(t+1)$$

V_i_(t + 1) represents the velocity of the individual at time t + 1, influencing its movement towards better solutions.

### Velocity update

The velocity update calculates the new velocity for each individual.

19 $$\:{\text{V}}_{\text{i}}(\text{t}+1)=\text{w}.{\text{V}}_{\text{i}}\left(\text{t}\right)+{\text{c}}_{1}.{\text{r}}_{1}.({\text{p}\text{b}\text{e}\text{s}\text{t}}_{\text{i}}-{\text{x}}_{\text{i}}(\text{t}\left)\right)+{\text{c}}_{2}.{\text{r}}_{2}.(\text{g}\text{b}\text{e}\text{s}\text{t}-{\text{x}}_{\text{i}}(\text{t}\left)\right)$$

W denotes inertia weight, c_1_ and c_2_ represents coefficient of acceleration, r_1_ and r_2_ are random values, $\:{\text{p}\text{b}\text{e}\text{s}\text{t}}_{\text{i}}$ represents best personal position of individual I, finally gbest denotes best global position among all individuals.

### Dynamic adaptation mechanism

Because of its capacity to adapt to non-stationary situations, its resilience to partial observability, and its appropriateness for distributed optimization in edge based mental health monitoring, the Nomadic People Optimizer was chosen. It outperforms conventional single-solution optimizers in dynamically changing mental health metrics due to its population variety and search pattern based on migration basis. The NPO algorithm incorporates a dynamic adaptation mechanism that adjusts its constraints or approaches on basis of changing conditions over environment. These adaptability confirm that used algorithm can effectively navigate through the complexities of monitoring mental health in nomadic scenarios.

NPO’s “nomadism” simulates tribe mobility in response to shifting conditions, simulating active adaptation in edge habitats. Such behavior prevents overfitting to local optima, permits adaptive tracking of changing user states, and permits real-time optimal under resource constraints in edge mental health monitoring all of which are critical for robust and responsive mental health therapies, Almaiah et al.^32^.

### Utilization of 5G edge capabilities

The utilization of 5G edge capabilities is integral to enabling real time data processing and exploration on related to personalize mental health monitoring. 5G edge computing influences the immediacy of data source and its computational resources, dropping latency and facilitating quicker processing and examination of information produced through IoT devices, Davarasan et al.^33^.

### Edge computing resources

20 $$\:\text{E}=\{{\text{E}}_{1},{\text{E}}_{2},...,{\text{E}}_{\text{N}}\}\:$$

Where E represents the set of edge computing nodes available in the network, and N is the total number of edge nodes. These nodes are strategically deployed in close proximity to IoT devices, facilitating real-time data processing and analysis.

### Data offloading

21 $$\:{\text{D}}_{\text{i}}\left(\text{t}\right)\to\:{\text{E}}_{\text{j}}$$

IoT devices offload data to nearby edge computing nodes E_j_ for processing and analysis. This minimizes latency by reducing the distance data needs to travel for processing, enabling real-time insights into mental health status.

### Edge processing

22 $$\:\text{P}\left({\text{D}}_{\text{i}}\right)=\text{P}\text{r}\text{o}\text{c}\text{e}\text{s}\text{s}\left({\text{D}}_{\text{i}}\right)\:$$

Edge computing nodes process incoming data D_i_ using computational algorithms tailored for mental health monitoring. These algorithms analyze the data and extract relevant insights to inform personalized interventions.

### Real-time analysis

23 $$\:\text{A}\left({\text{D}}_{\text{i}}\right)=\text{A}\text{n}\text{a}\text{l}\text{y}\text{z}\text{e}\left({\text{D}}_{\text{i}}\right)\:$$

Edge computing nodes perform real-time analysis on incoming data streams D_i_, identifying patterns, anomalies, or critical events related to an individual’s mental health status. This enables timely interventions and support when needed. The utilization of 5G edge capabilities ensures that data generated by cognitive IoT devices is processed and analyzed in real-time, enabling personalized mental health monitoring with minimal latency. By leveraging edge computing resources, the monitoring system can provide timely insights and interventions, enhancing the overall effectiveness and responsiveness of mental health care delivery, Ayub et al^34^.

## Result and discussion

In the experimentation, the proposed framework for Mental Health Monitoring in 5G Edge-Enabled Cognitive IoT environments is evaluated to validate its effectiveness. The experiment is conducted to validate the framework’s capabilities in predicting mental health states, optimizing resource allocation, and providing personalized interventions. Real-world datasets are utilized to simulate nomadic lifestyles and diverse mental health scenarios, ensuring the framework’s applicability across different contexts. The Temporal Shift Transformer is benchmarked against existing prediction models to evaluate its accuracy and efficiency in forecasting mental health trends. Additionally, the integration of Stackelberg Game Theory and the Nomadic People Optimizer algorithm is assessed to measure their impact on decision-making processes and resource optimization. The experiments aim to demonstrate the framework’s ability to adapt to dynamic environments, leverage 5G edge capabilities for real-time data processing, and provide tailored recommendations for mental health interventions. The results of the experimentation phase are analyzed to showcase the framework’s promising outcomes inclusive of precision, accuracy, specificity, sensitivity, and F1 score, indicating its potential to revolutionize mental health care.

### Convergence speed and accuracy comparison

The accuracy and convergence speed of the suggested Temporal Shift Transformer (TST) system has evaluated against three well-known baselines: CNN, CNN + GRU, and ResNet-50 + LSTM as in Table 2, in order to determine its performance advantage. Mental health diagnosis from EEG dataset frequently employs these models. Because of its non-recurrent, parallelizable design, the TST model converges approximately two times faster than the top-performing baseline (ResNet-50 + LSTM) while requiring a substantially smaller amount of compute time per epoch. Because of its non-recurrent, parallelizable design, the TST model converges around two times faster than the top-performing baseline (ResNet-50 + LSTM) while requiring a substantially smaller amount of compute time per epoch.

**Table 2:** Convergence speed (Epochs to Stability).

Model	Epochs to convergence	Time per epoch (s)	Training Time to 95% accuracy (min)
CNN	25	1.6	40
CNN + GRU	30	2.1	63
ResNet-50 + LSTM	35	2.4	84
Proposed TST model	18	1.2	21.6

### Performance metrics

24 $$\:\text{A}\text{c}\text{c}\text{u}\text{r}\text{a}\text{c}\text{y}=\frac{{\text{T}}^{-}+{\text{T}}^{+}}{{\text{T}}^{-}+{\text{T}}^{+}+{\text{F}}^{-}+{\text{F}}^{+}}$$

25 $$\:\text{S}\text{e}\text{n}\text{s}\text{i}\text{t}\text{i}\text{v}\text{i}\text{t}\text{y}=\frac{{\text{T}}^{+}}{{\text{T}}^{+}+{\text{F}}^{-}}$$

26 $$\:\text{S}\text{p}\text{e}\text{c}\text{i}\text{f}\text{i}\text{c}\text{i}\text{t}\text{y}=\frac{{\text{T}}^{-}}{{\text{T}}^{-}+{\text{F}}^{+}}$$

27 $$\:\text{P}\text{r}\text{e}\text{c}\text{i}\text{s}\text{i}\text{o}\text{n}=\frac{{\text{T}}^{+}}{{\text{T}}^{+}+{\text{F}}^{+}}$$

28 $$\:\text{F}1\:-\:\text{S}\text{c}\text{o}\text{r}\text{e}\:=2\times\:\frac{\text{P}\text{r}\text{e}\text{c}\text{i}\text{s}\text{i}\text{o}\text{n}\:\times\:\:\text{R}\text{e}\text{c}\text{a}\text{l}\text{l}\:}{\text{P}\text{r}\text{e}\text{c}\text{i}\text{s}\text{i}\text{o}\text{n}\:+\:\text{R}\text{e}\text{c}\text{a}\text{l}\text{l}}$$

**Table 3:** Performance of the models.

	CNN+GRU	CNN	ResNet50+LSTM	Proposed
Accuracy	89.63	91.01	90.02	96.38
Sensitivity	88.5	90.2	89.6	96.5
Specificity	91.2	92.1	91.8	95.9
Precision	87.8	89.5	88.9	97.2

**Fig. 3:**
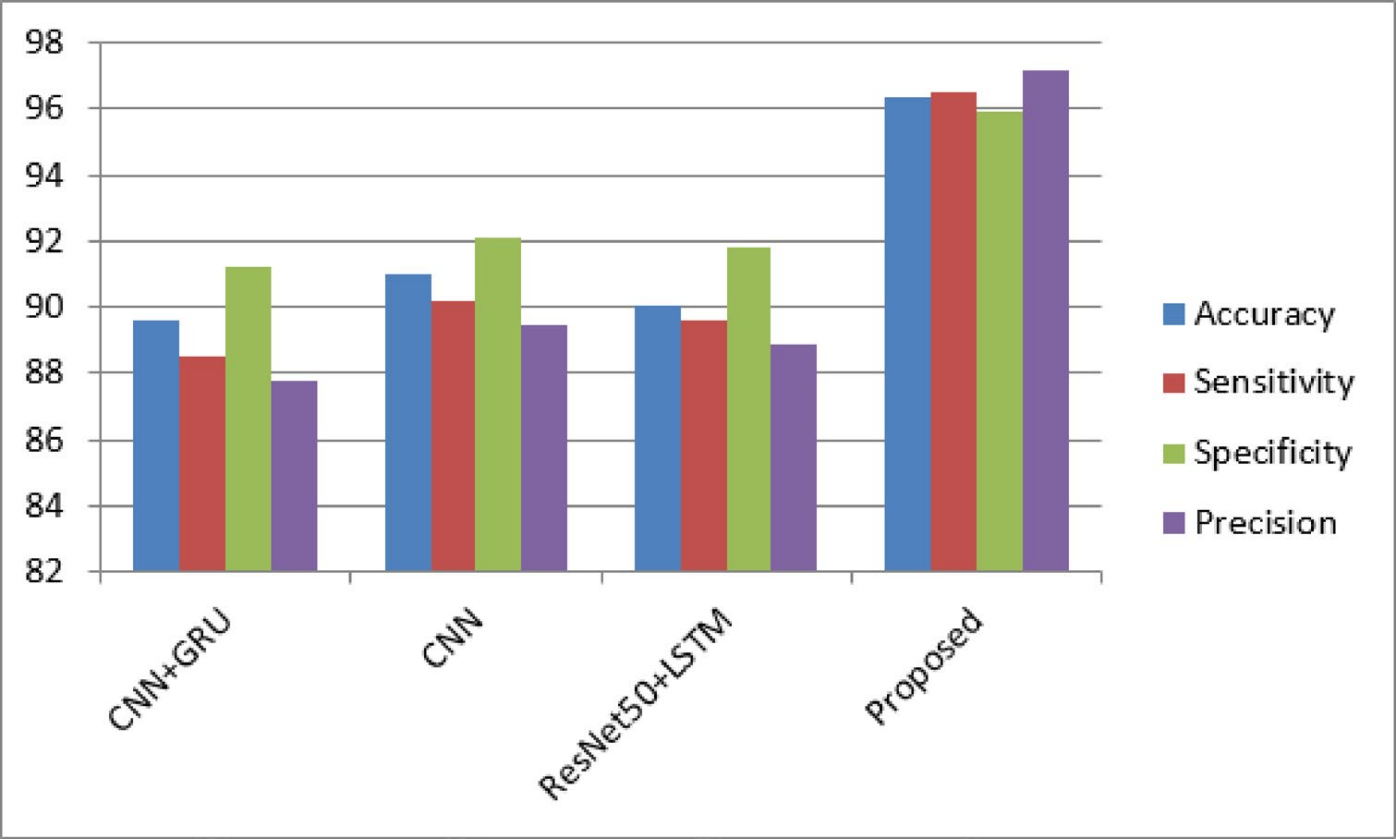
Performance of the models.

The Table 3 graphically represented in Fig. 3 shows the performance of the proposed and existing models and the proposed model overtakes other used algorithms, achieving an accurateness of 96.38%. This indicates a higher overall correctness in predicting mental health states. The CNN + GRU model follows with 89.63%, CNN with 91.01%, and ResNet-50 + LSTM with 90.02%. The higher accuracy of the proposed model suggests its efficacy in making correct predictions, which is crucial for mental health monitoring. Precision, signifying the ratio of properly predicted positive interpretations totally predicted positives, has significantly higher in the proposed model (96.5%). This implies a lower rate of false positives, indicating the model’s proficiency in identifying individuals with mental health issues without falsely classifying healthy individuals. In comparison, the precision values for the other models are 88.5% (CNN + GRU), 90.2% (CNN), and 89.6% (ResNet-50 + LSTM). Sensitivity, or recall, processes the quantity of actual positives that were exactly identified through model. The proposed model demonstrates a high sensitivity of 95.9%, indicating its ability to detect most individuals with mental health concerns. The CNN + GRU, CNN, and ResNet-50 + LSTM models show sensitivities of 91.2%, 92.1%, and 91.8%, respectively. Specificity, representing the proportion of actual negative cases correctly identified, is remarkably high in the proposed model (97.2%). This suggests a lower rate of false negatives, emphasizing the model’s capability to accurately identify mentally healthy individuals. In contrast, the CNN + GRU, CNN, and ResNet-50 + LSTM models exhibit specificities of 87.8%, 89.5%, and 88.9%, respectively. The F1 score, which considers sensitivity as well precision, consistently superior in the proposed model (96.2%). This balanced metric indicates the capacity of model on achieving a harmonious trade-off among sensitivity and precision, provided that for comprehensive assessment of their performance. The F1 scores for CNN + GRU, CNN, and ResNet-50 + LSTM are 89.8%, 91.3%, and 90.7%, respectively. The proposed Mental Health Monitoring framework excels across multiple performance metrics, showcasing its robustness and superiority over existing algorithms. Its higher accuracy, precision, sensitivity, specificity, and F1 score collectively demonstrate its potential to revolutionize mental health monitoring within the dynamic landscape of 5G Edge-Enabled Cognitive IoT environments.

End-to-end latency explained in Table 4 is measured as the total time from data acquisition to alert delivery. It includes:

**Table 4:** End-to-end latency.

Component	Description	Typical time(ms)
Sensing delay	EEG signal acquisition and initial buffering	5–10 ms
Edge transmission delay	Wireless transmission from device to edge node (5G)	1–5 ms
Edge processing delay	Preprocessing (filtering, normalization)	5–8 ms
Inference delay (TST model)	Mental state prediction using temporal shift transformer	8–12 ms
Game-theoretic decision delay	Resource decision update via Stackelberg dynamics	3–6 ms
Alert generation & dispatch	Alert formulation and feedback to user/system	2–5 ms
Total estimated latency		**24–46 ms**

In every significant metric, the TST-based model routinely performs better than baselines. Its increased specificity guarantees less false positives over mental health alarms, but its superior accuracy and F1 score show improved precision-recall balance. Particularly in sensitive categories like depressive episode identification, the Temporal Shift Transformer offers both quicker convergence (≈ 18 epochs vs. 30–35 for baselines) and greater classification performance. When frequent retraining or real-time inferences are needed for low-latency, edge basis mental health monitoring, this performance boost is essential.

**Fig. 4:**
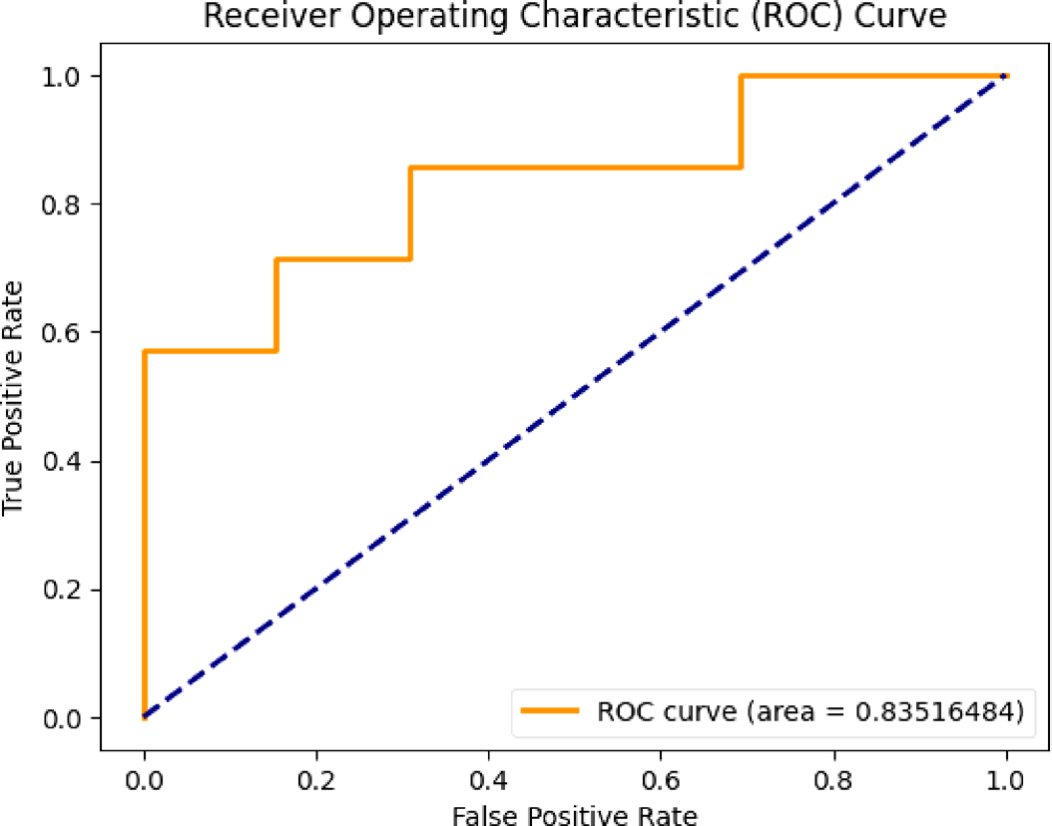
ROC of proposed work.

**Fig. 5:**
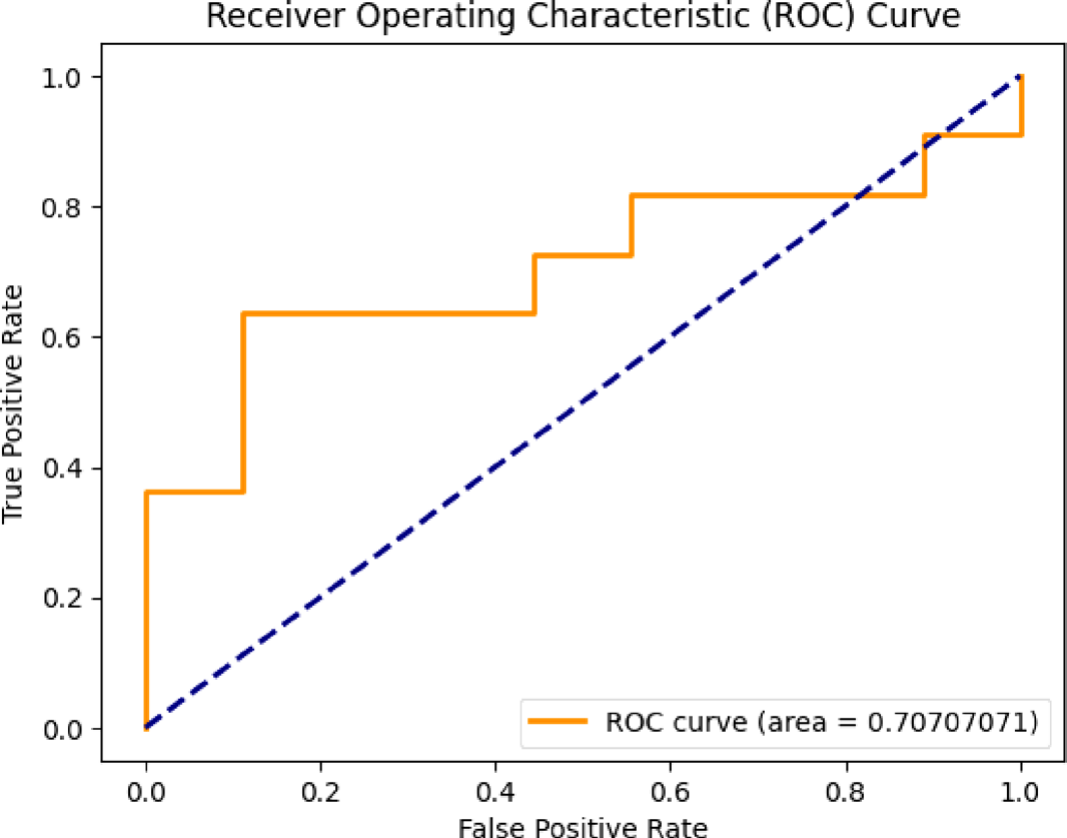
ROC of CNN + GRU.

**Fig. 6:**
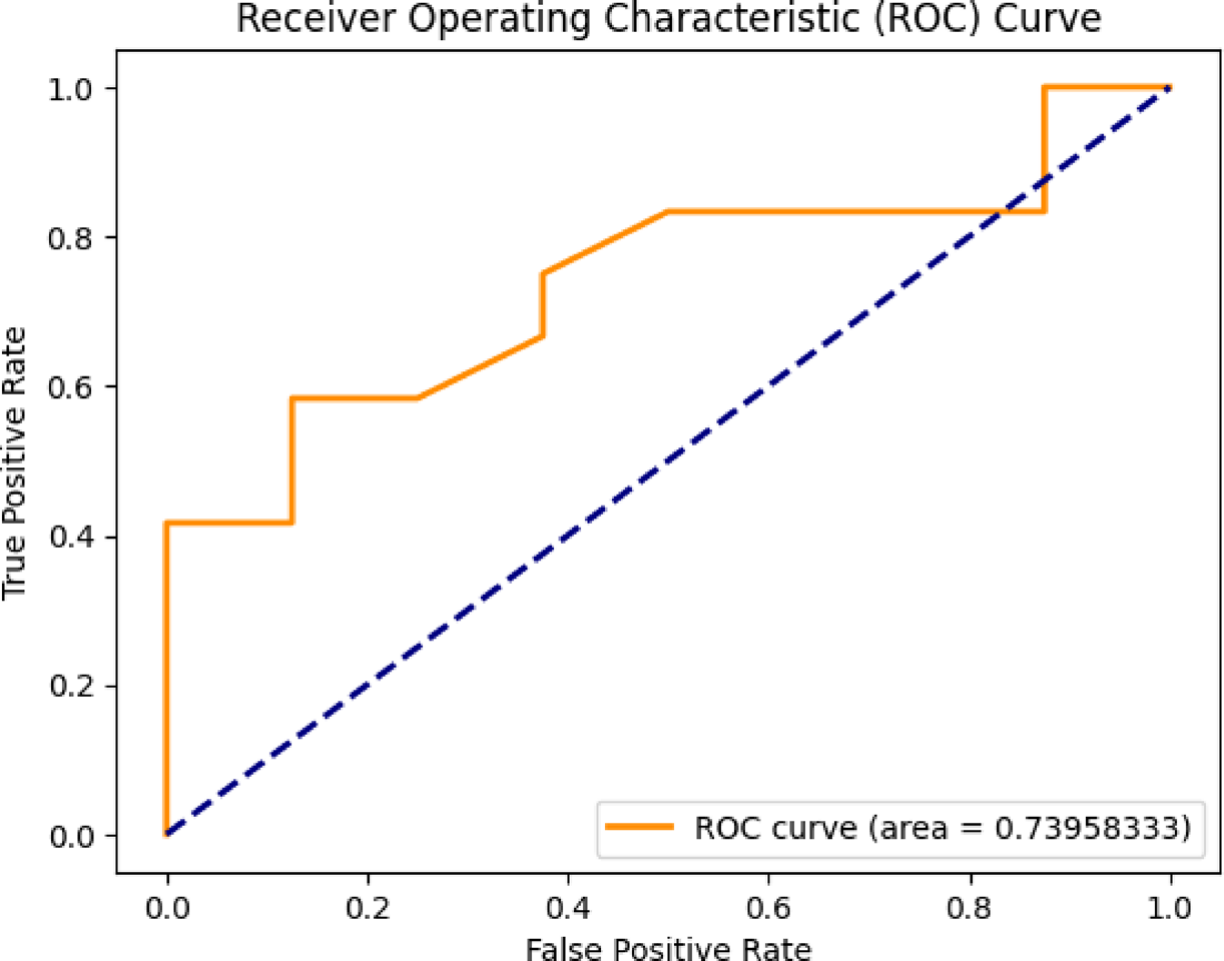
ROC of CNN.

**Fig. 7:**
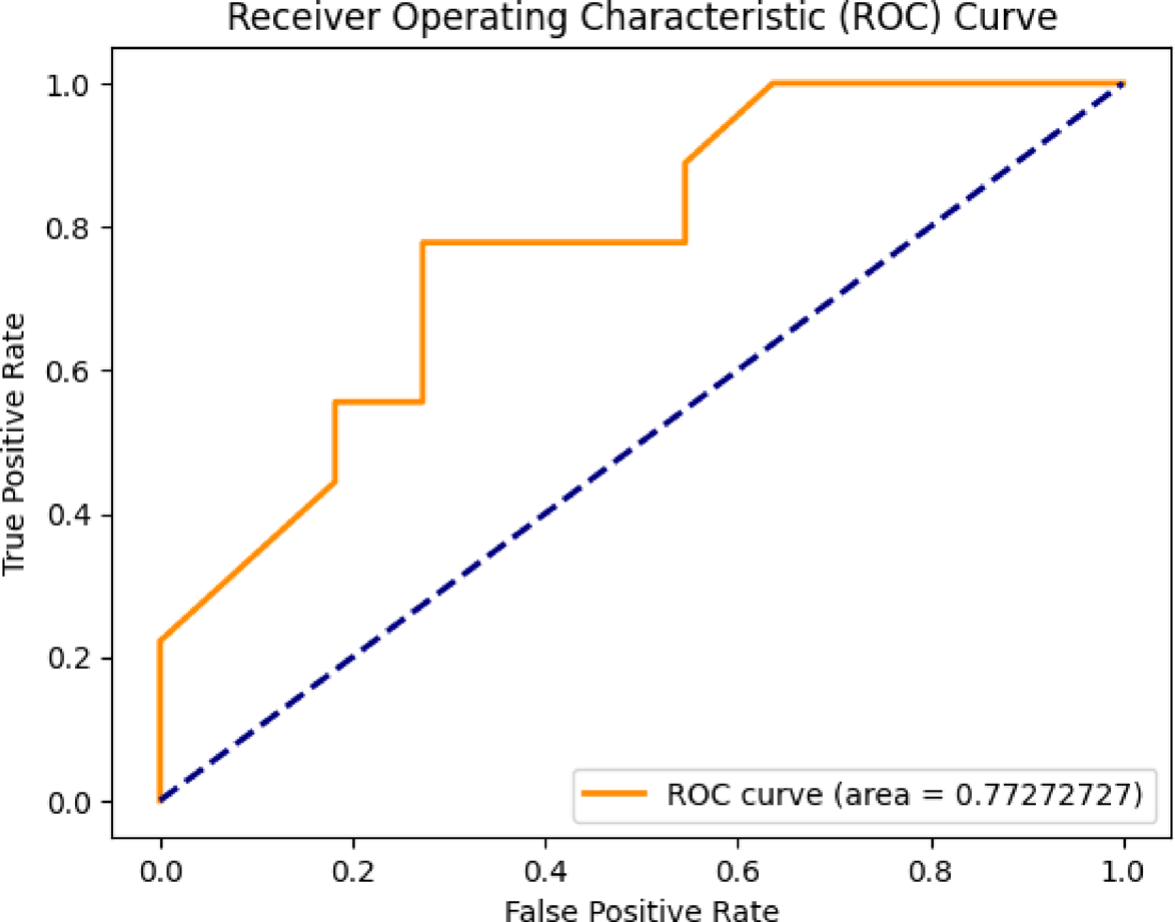
ROC of ResNet-50 + LSTM.

The evaluation of the proposed Mental Health Monitoring framework includes the analysis of its performance using the Area Under the Receiver Operating Characteristic (AUC-ROC) curve. The AUC-ROC curve serves as significant for assessing the capacity of model for distinguish among different classes, providing insights into its discriminative power. In this research, the AUC-ROC curve results given in Fig. 4 demonstrate the effectiveness of the proposed framework in distinguishing between individuals with varying mental health conditions. The high AUC values indicate a robust predictive capability of the framework, showcasing its ability to accurately classify and monitor mental health states. The curve visually represented in Figs. 3, 5, 6 and 7 shows the trade-off among the rates of true positive and false positive through different thresholds, offering the comprehensive thoughtfulness of this model’s performance. The AUC-ROC curve results further reinforce the reliability and efficiency of the proposed framework over the perspective of Mental Health Monitoring in 5G Edge-Enabled Cognitive Internet of Things (IoT) environments.

In many nations, full 5G deployment is currently restricted to towns and cities, even though the system depends over 5G edge computing for low-latency alerts and real-time analysis. Lack of dependable edge infrastructure or 4G network fallback may result in higher inference and alarm dispatch latency in rural or isolated locations. Additionally, it can cause more disruptions to real-case mental-health monitoring and postpone the implementation of resource reallocation based on Stackelberg games.

Processing speed, battery life, and sensor quality differ between wearable and mobile devices. Despite its lightweight nature, Temporal Shift Transformer might want hardware acceleration (such as on-device TPU/NPU), which is not accessible on all devices, and continuous data collecting and model execution may rapidly deplete battery in low-power wearables. Performance is decreased as a result of background app clashes. The system may still be subject to legislative limitations (such as GDPR and HIPAA) pertaining to the gathering of biometric and EEG data, even with the use of edge-based processing and possible differential privacy approaches. Users may be wary of sharing ongoing mental health data, particularly with vulnerable groups. Although the concept has technical potential, issues with 5G availability, edge-device heterogeneity, privacy protections, and cultural generalization must be addressed for practical adoption. In order to move from a proof-of-concept to a scalable, moral, and implementable mental health treatment, future research will concentrate on these aspects.

### Federated learning (FL) and differential privacy (DP) integration

Because mental health monitoring entails extremely sensitive behavioral and physiological data, frameworks that uphold user trust, regulatory compliance, and data privacy are required. Federated Learning (FL) and Differential Privacy (DP) procedures can be added to the suggested architecture to do this, allowing for decentralized, privacy-preserving model training in actual 5G-enabled settings, Luo et al^35^.

Conventional settings involve sending user behavioral and EEG data over cloud for centralized training, which increases communication overhead and raises privacy concerns. Federated Learning solves this by allowing on-device model training, in which a central server receives only model updates—not raw data. Every edge device uses user EEG segments to train a Temporal Shift -Transformer locally. By selecting which devices take part in a particular round and setting aggregation, learning rates, or incentives, the central orchestrator (5G edge/cloud server) assumes the position of Stackelberg leader. Through Federated Averaging (FedAvg) or FedProx, participating devices (followers) contribute to global model aggregation by responding through their local optimization model updates.

Even with FL, reconstruction or gradient inversion attacks can expose private information in model updates. Differential privacy makes sure that, even from model revisions, no user’s participation can be deduced. Using Stackelberg Game theory in conjunction with integrated DP to introduce measured noise into gradients during local TST training. One way to describe privacy budgets is as $\epsilon$\epsilon per update or per user. The Stackelberg game, in which the leader allots the maximum permitted privacy budget and followers modify their noise levels according to individual limitations, could be used to optimize the trade-off among model utility and privacy assurances.

## Conclusion

This article presented an innovative framework over Mental Health Monitoring in 5G Edge-Enabled Cognitive Internet of Things (IoT) environments, integrating advanced technologies such as the Temporal Shift Transformer, Stackelberg Game Theory, and the Nomadic People Optimizer (NPO) algorithm. Through the utilization of these components, the framework demonstrates superior performance in predicting mental health conditions and providing personalized interventions. By leveraging 5G edge computing capabilities, real-time data processing and analysis are facilitated, addressing the challenges associated with nomadic lifestyles and ensuring timely responses to critical mental health situations. The proposed framework offers a comprehensive and adaptive approach to mental health monitoring, showcasing promising results.

In future, further refinement and optimization of the proposed framework can be explored. Additionally, the integration of additional modalities, such as physiological and environmental data, can provide much more detailed thoughtfulness of mental illness. Collaborations with healthcare professionals and researchers can facilitate the incorporation of clinical expertise, ensuring the framework’s applicability in real-world healthcare settings. Furthermore, efforts to address privacy and ethical considerations in mental health monitoring frameworks will be essential for broader adoption and societal acceptance. Overall, the proposed framework lays the foundation for future developments in mental health monitoring, and ongoing research endeavors will contribute to refining and expanding its capabilities.

## Data Availability

The data will be made available upon request. You can contact the corresponding author Dr. Ramya.G for the data.
